# Single-cell measurements and modelling reveal substantial organic carbon acquisition by *Prochlorococcus*

**DOI:** 10.1038/s41564-022-01250-5

**Published:** 2022-11-03

**Authors:** Zhen Wu, Dikla Aharonovich, Dalit Roth-Rosenberg, Osnat Weissberg, Tal Luzzatto-Knaan, Angela Vogts, Luca Zoccarato, Falk Eigemann, Hans-Peter Grossart, Maren Voss, Michael J. Follows, Daniel Sher

**Affiliations:** 1 Department of Earth, Atmospheric and Planetary Sciences, Massachusetts Institute of Technology, Cambridge, MA USA; 2 Department of Marine Biology, Leon H. Charney School of Marine Sciences, University of Haifa, Haifa, Israel; 3 Leibniz-Institute for Baltic Sea Research, Warnemuende, Germany; 4 Department of Experimental Limnology, Leibniz-Institute of Freshwater Ecology and Inland Fisheries, Stechlin, Germany; 5 Institute of Biochemistry and Biology, Potsdam University, Potsdam, Germany

**Keywords:** Biogeochemistry, Water microbiology, Biogeochemistry, Ecological modelling

## Abstract

Marine phytoplankton are responsible for about half of the photosynthesis on Earth. Many are mixotrophs, combining photosynthesis with heterotrophic assimilation of organic carbon, but the relative contribution of these two lifestyles is unclear. Here single-cell measurements reveal that *Prochlorococcus* at the base of the photic zone in the Eastern Mediterranean Sea obtain only ~20% of carbon required for growth by photosynthesis. This is supported by laboratory-calibrated calculations based on photo-physiology parameters and compared with in situ growth rates. Agent-based simulations show that mixotrophic cells could grow tens of metres deeper than obligate photo-autotrophs, deepening the nutricline by ~20 m. Time series from the North Atlantic and North Pacific indicate that, during thermal stratification, on average 8–10% of the *Prochlorococcus* cells live without enough light to sustain obligate photo-autotrophic populations. Together, these results suggest that mixotrophy underpins the ecological success of a large fraction of the global *Prochlorococcus* population and its collective genetic diversity.

## Abstract (web summary)

Single-cell measurements and modelling show that the cyanobacterium *Prochlorococcus* obtain most of the carbon required for growth from mixotrophy, extending their ecological range and impact in the water column.

## Main

Photosynthesis by phytoplankton provides most of the energy and fixed carbon that support marine food webs and carbon reservoirs^1^. However, few phytoplankton are strictly photo-autotrophic^2^. Many phytoplankton also utilize dissolved organic matter, taking up particulate detrital organic matter or preying upon other living cells and even harvesting organelles^2^. Mixotrophic lifestyles (osmotrophy, hereafter), in which cells both fix carbon and use exogenously available organic carbon, may enhance fitness, for example when the relative availability of inorganic resources differs from physiological demands (for example, light intensity is low but inorganic nutrients are abundant)^3^. Mixotrophy can also save energy and can reduce resources available to competitors^2^. Despite the potential importance of mixotrophy to phytoplankton life history, the contribution of heterotrophic carbon assimilation to phytoplankton growth is not well quantified^4^. Simulations suggest that mixotrophy may be a globally important carbon source for phytoplankton^5^, but this prediction is currently difficult to quantitatively test with empirical data. One reason is that dissolved organic carbon (DOC) in the oceans constitutes an extremely complex mixture of compounds^6,7^, most of which are uncharacterized. This means that uptake measurements using specific organic carbon sources (for example, glucose and amino acids)^8,9^ do not represent the entire available DOC pool and may underestimate the actual DOC uptake rates and, hence, mixotrophy of major phytoplankton species^10^.

*Prochlorococcus* are the most abundant phototrophic cells on Earth, actively growing at depths ranging from the ocean surface down to the base of the photic zone (~160 m) (ref. ^11^). Across these depths, photosynthetically available radiation (PAR) varies over three to four orders of magnitude, a challenge that the diverse *Prochlorococcus* lineage faces using a variety of adaptations in their photosynthetic apparatus^11,12^. These adaptations have led to the diversification of *Prochlorococcus* into high light (HL)- and low light (LL)-adapted clades^11,12^. In addition, *Prochlorococcus* are mixotrophs, able to uptake dissolved organic compounds such as glucose^8^, pyruvate^13^, amino acids^9^, nucleotides^10^ and perhaps dimethylsulfoniopropionate^14,15^. Yet, to what extent DOC uptake can supplement or replace photosynthetically fixed carbon for respiration and/or growth in this globally abundant lineage is still unknown^10^. Available evidence suggests that, while mixotrophy helps *Prochlorococcus* survive limited periods of darkness, axenic cells die after ~1 week if not exposed to light^13,16^, indicating that light harvesting, and possibly photosynthesis, is probably obligate.

In this Article, we take a multi-faceted approach to evaluate the contribution of heterotrophic carbon assimilation to *Prochlorococcus* in the oceans. We first use isotopic measurements to quantify photosynthesis and N uptakes in wild *Prochlorococcus* populations at the base of the photic zone in the Mediterranean Sea. Then we compare observed growth rates from the Pacific Ocean with purely photo-autotrophic growth rates simulated by a laboratory-calibrated photo-physiological model. We also use an individual-based model to illustrate how mixotrophy provides a fitness advantage and deepens the nutricline. Finally, we use time-series observations of vertical profiles of *Prochlorococcus* ecotypes in subtropical gyres to show that several clades rely extensively on mixotrophic carbon assimilation. Overall, these results suggest that up to a quarter of depth-integrated carbon assimilation by *Prochlorococcus* originates from DOC, with implications for global C cycles, and that mixotrophy is essential to support a substantial fraction of *Prochlorococcus* diversity.

## Results

### Carbon and nitrogen uptake at the base of the photic zone

**Fig. 1:**
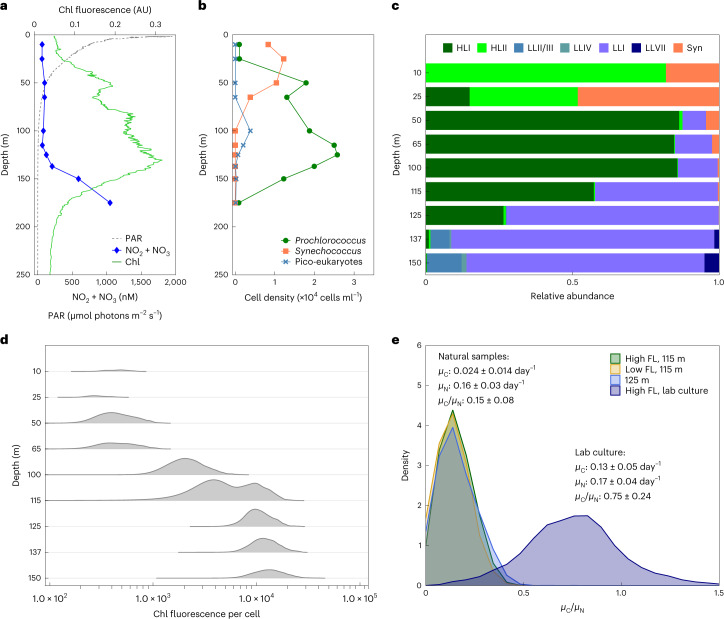
Nutrient uptake of naturally occurring *Prochlorococcus* populations at the Eastern Mediterranean Sea. **a**, Depth profiles of PAR, NO_2_ + NO_3_ and chlorophyll (Chl). **b**, Phytoplankton cell counts using flow cytometry. **c**, Relative abundance of different *Prochlorococcus* clades across the water column, determined by ITS sequencing (HLI denotes high-light-adapted clade I, HLII denotes high-light-adapted clade II, LLI denotes low-light-adapted clade I, LLII/III denotes low-light-adapted clades II and III, LLIV denotes low-light-adapted clade IV, LLVII denotes low-light-adapted clade VII, Syn denotes *Synechococcus*). **d**, Density plots of *Prochlorococcus* per-cell chlorophyll fluorescence (FL). Note the change in chlorophyll fluorescence (**d**) without a concomitant change in population structure (**c**) between 65 m and 100 m. Note also the presence of LL clades above 115 m and HL clades at 125 m (**c**) while a double population is observed only at 115 m (**d**). The circles in **d** represent the populations sorted and analysed by NanoSIMS, and are colour-coded as in **e**. **e**, Density plots of the ratios of C-specific C uptake rate (*μ*_C_) to N-specific N uptake rate (*μ*_N_) from NanoSIMS analysis of each sorted subpopulation from 115 m, the single population from 125 m, and lab cultures. The numbers of cells measured in each population are 45 (LL 115 m), 49 (HL 115 m), 55 (125 m) and 489 (lab culture). The scatter plots and gates used for these analyses are shown in Extended Data Fig. 1. Source data

To evaluate the relative contributions of photosynthesis and heterotrophic carbon uptake in a natural *Prochlorococcus* population from the base of the photic zone, where light may be limiting, we assess the *Prochlorococcus* population structure and per-cell activity during late summer in the ultra-oligotrophic Eastern Mediterranean Sea^17^. At the time of sampling, the water column was highly stratified, nutrients were depleted down to around 140 m, and a prominent deep chlorophyll maximum (DCM) was observed at depth of ~115 m (Fig. 1a). *Prochlorococcus* were the numerically dominant phytoplankton below the surface (Fig. 1b), and could be divided into two populations on the basis of the per-cell fluorescence: a low-fluorescence population from the surface to 115 m and a high-fluorescence population from 115 m to 150 m, with an overlap at 115 m (Fig. 1c,d). The shift in the per-cell chlorophyll fluorescence in *Prochlorococcus* with depth is commonly observed^18–20^, and is usually attributed to a change in the genetic composition of the population, from HL-adapted cells (low fluorescence) to LL-adapted (high fluorescence) ones^19^. However, phenotypic heterogeneity (acclimation) can also contribute to this phenomenon^21^, and indeed amplicon sequencing of the internal transcribed spacer between the 16S and 23S genes (ITS)^21,22^ revealed a gradual transition from HL to LL clades around the DCM, suggesting both genotypic and phenotypic shifts with depth (Fig. 1c). The flow cytometry and genetic data are both consistent with previous studies^21,23^, and suggest that the water column had been relatively stable for at least 3–4 days before sampling^20^. Notably, the light intensity at the DCM (~3–5 µmol photons m^−2^ s^−1^ at 115–125 m depth during the afternoon, Fig. 1a) is potentially enough under laboratory conditions to support the growth of some LL strains but not sufficient for active growth of most HL strains^24^. Since HL cells account for >50% of the *Prochlorococcus* population at 115 m and about 25% at 125 m, this suggests that a considerable fraction of the *Prochlorococcus* cells in these samples are living under conditions where laboratory cultures cannot grow purely autotrophically (Fig. 1c).

We next measured the uptake of ^13^C-labelled bicarbonate (representing C fixation through photosynthesis) and of ^15^N-labelled ammonium (representing nitrogen uptake) in single *Prochlorococcus* cells from the DCM, using nanoscale secondary ion mass spectrometry (NanoSIMS). Essentially, all of the *Prochlorococcus* cells at 115 and 125 m depth were active (photosynthesized and took up NH_4_). The observation that essentially all of the *Prochlorococcus* cells in natural samples are active is consistent with a similar study in the North Pacific^25^, and suggests that dead or chlorotic cells observed in laboratory cultures^13,26^ may be relatively rare in nature, at least during mid-day at the DCM. Nevertheless, the per-cell photosynthesis rates at these depths were not sufficient to support the growth rates indicated by the nitrogen-specific nitrogen uptake rates, even though the uptake experiments were performed when light intensity was maximal (Fig. 1e). Previous studies from multiple oceanic regions based on cell cycle analysis and on ^14^C incorporation into divinyl-chlorophyll indicate that *Prochlorococcus* cells at depths of 100–150 m replicate every 4–7 days (a growth rate of 0.14–0.25 day^−1^) (refs. ^27–30^). However, the observed C-specific C uptake rate (*μ*_C_) was only ~0.024 day^−1^, too low to support these expected growth rates, while the observed N-specific N uptake rate (*μ*_N_) was ~0.16 day^−1^, indicating a doubling time of ~6 days. Furthermore, *μ*_C_/*μ*_N_ was only ~0.15 in the field, much lower than normal cells, which are expected to be ~1 (*μ*_C_ ≈ *μ*_N_). Indeed, *μ*_C_/*μ*_N_ in lab-cultured *Prochlorococcus* was ~0.75 (Fig. 1e), and was ~1 in surface samples from North Pacific Subtropical Gyre and in the California Current System^25^. Taken together, these quantitative observations suggest that >80% of the C required for the expected growth rate of these *Prochlorococcus* cells at the DCM must come from non-photosynthetic sources.

### Evaluation of potential growth rate profiles

**Fig. 2:**
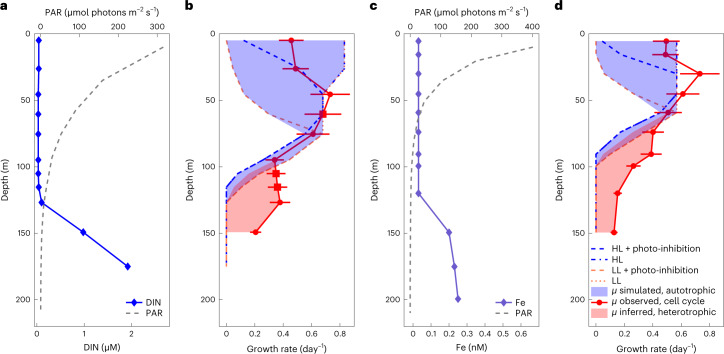
Simulated growth rates at the HOT and EqPac. **a**,**c**, Observations of PAR and dissolved inorganic nitrogen (DIN) at the HOT (July 1994, **a**) and of PAR and dissolved iron (Fe) at the EqPac (April 1992, **c**). **b**,**d**, Simulated autotrophic growth rates (dashed blue and orange lines) and observed growth rates (red solid line with dots, derived from cell cycle analysis; red squares are linear-interpolated data points; data from Liu et al.^32^ and Vaulot et al.^31^) at HOT (**b**) and EqPac (**d**). The blue and magenta dashed lines represent the simulated autotrophic growth rates using HL and LL photosynthesis parameterizations with/without photo-inhibition, respectively (for more information, see Methods). The blue shading represents the extreme ranges of the simulated autotrophic growth rates in the four scenarios above. The red shading represents the inferred heterotrophic growth rate as the difference between simulated autotrophic growth rate and observed growth rate. The measured growth rates include both autotrophic and heterotrophic growth, and are representative of other depth profiles from the same studies where growth was observed at depths of at least 150 m. The error bars represent a 19% error (*n* = 11 biologically independent samples) of observed growth rate at HOT and EqPac according to Vaulot et al.^31^. The Fe concentrations above 120 m shown in **c** were reported as less than 0.03 nmol l^−1^ (below the limit of detection^37^), but we use 0.03 nmol l^−1^ for the calculation. Source data

**Extended Data Fig. 2:**
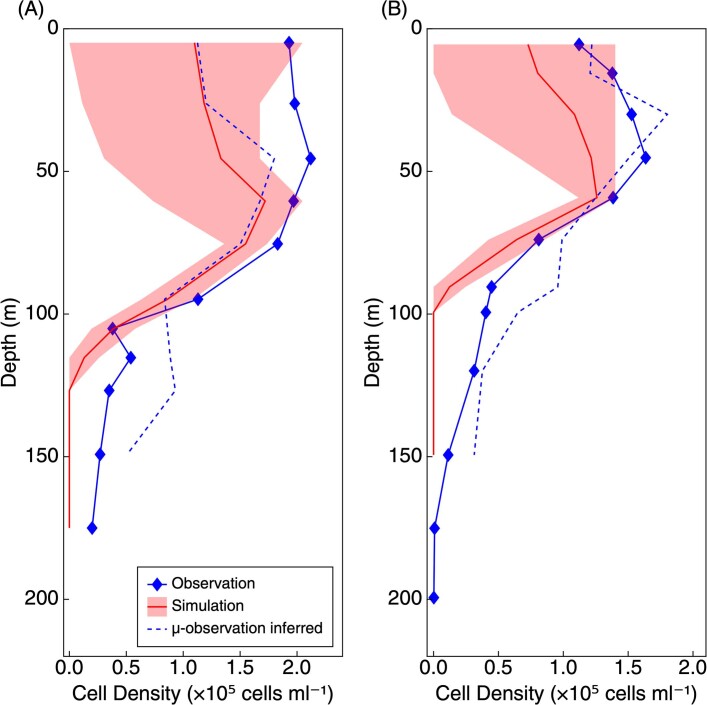
Simulated and observed cell densities at Station ALOHA (panel A) and equatorial Pacific (panel B). The blue lines represent observed cell densities of Prochlorococcus. The blue dashed lines represent the cell densities derived from observed growth rates based on Equation (4). The red lines with red shapes represent simulated cell densities. Source data

Our Mediterranean samples suggest that a large fraction of carbon assimilated by *Prochlorococcus* in the deeper reaches of the photic zone is of organic origin. We can test this interpretation by evaluating the carbon-specific photosynthetic carbon fixation rate (day^−1^) and comparing it with in situ observed growth rates (day^−1^) based on cell cycle analysis^31,32^. If the photosynthesis rate is smaller than the observed growth rate, we interpret the difference as heterotrophically supported growth. To evaluate the carbon-specific photosynthesis rate (day^−1^) as a function of depth, we employed the standard representation of the photosynthesis–irradiance relationship^33^ (equation (4) and Methods) with laboratory-calibrated parameter values, driven by observed photon flux densities. Since chlorophyll:C ratios were not measured, they were evaluated as a function of light intensity and growth rate using a laboratory-calibrated model of macromolecular allocation^34^ that also evaluates the maximum growth rate and assumes a fixed maintenance respiration rate (for details, see Methods). Thus equation (5) was used to evaluate the potential autotrophic growth rate. To bracket the range of possible photosynthesis rates at each depth, we used photosynthesis–irradiance parameters for HL and LL ecotypes, each acclimated at both HL and LL, from Moore and Chisholm^24^. In the upper photic zone, nutrients are limiting, rather than light. Thus, we also evaluated nutrient-specific nutrient uptake rates (day^−1^) as a function of depth using allometric scaling for fixed-nitrogen, phosphate and dissolved iron uptake rates^35,36^, driven by observed environmental concentrations. We assumed that the minimum of the specific carbon or nutrient uptake rates controls autotrophic growth rate at each depth. We compared vertical profiles of estimated autotrophic and observed growth rates for *Prochlorococcus* at two sites in the Pacific where appropriate datasets are available. Observed cell-cycle-based growth rates for *Prochlorococcus* require intensive sampling at high temporal resolution, and are not available from the Mediterranean. Thus, we used cell-cycle-based growth rate data from Vaulot et al.^31^ and Liu et al.^32^ in the Equatorial Pacific (EqPac, 0° N, 140° W) and North Pacific Subtropical Gyre (Hawaii Ocean Time-series (HOT), 22° 45′ N, 158° W; Station ALOHA), respectively. The concurrent photon fluxes and nutrient concentrations were available from an extensive biogeochemical survey (JGOFS EqPac)^37^ and time-series station (HOT)^38^ respectively (Fig. 2a,c, Supplementary Text and Extended Data Fig. 2).

The estimated, purely autotrophic growth rates were determined by the most limiting resource at each depth (Fig. 2b,d). Light and carbon fixation strongly limited the simulated autotrophic growth in the deeper region of the photic layer, while fixed nitrogen (HOT), iron (EqPac) and carbon fixation, due to photo-inhibition, were important near the surface (Fig. 2). While the observed growth rates at the surface were mostly within the ranges predicted from the photo-physiological parameters of HL and LL strains (blue shading in Fig. 2b,d), the model failed to resolve the observed growth rates below ~75–100 m at both stations. Rather, the model unequivocally suggests that photosynthesis alone cannot account for the observed division rates at depth. We interpret the differences between the modelled autotrophic and observed actual growth rates at depth (red shading) to infer the minimal rate of organic carbon assimilation of *Prochlorococcus*. The two Pacific stations represent very different physical and biogeochemical regimes, yet show similar qualitative structure. Mixotrophy appears to become important at different depths at the two stations (95 m at HOT and 60 m at EqPac) but at similar level of PAR (~15 µmol photons m^−2^ s^−1^, ~5% of surface PAR). Using observed cell densities^31,32^ and assumed cellular carbon quotas^39^, we estimated the vertically integrated autotrophic net primary production for *Prochlorococcus* to be ~0.35 g C m^−2^ day^−1^ at HOT and ~0.20 g C m^−2^ day^−1^ at EqPac, with vertically integrated heterotrophic contributions (based on the red shading in Fig. 2b,d) of ~0.075 g C m^−2^ day^−1^ at HOT and ~0.069 g C m^−2^ day^−1^ at EqPac. In other words, assimilation of organic carbon is inferred to support ~18% of total *Prochlorococcus* biomass production at HOT and ~25% at EqPac. Furthermore, organic carbon uptake contributes ~80% at HOT and 54% at EqPac of the total production below the depth where the contribution of mixotrophy is greater than photosynthesis, broadly consistent with the isotopic inference from the deep photic zone in the Mediterranean. We note that this model does not take into account exudation of organic carbon by *Prochlorococcus*, which is not well constrained experimentally and would probably reduce the inferred growth rates at the surface^40–43^. Indeed, mixotrophy (uptake of glucose and amino acids) has been observed in surface *Prochlorococcus*^9,10^, suggesting that our estimate provides a lower bound of the contribution of mixotrophy to integrated *Prochlorococcus* production.

### Simulations in a dynamic water column

**Fig. 3:**
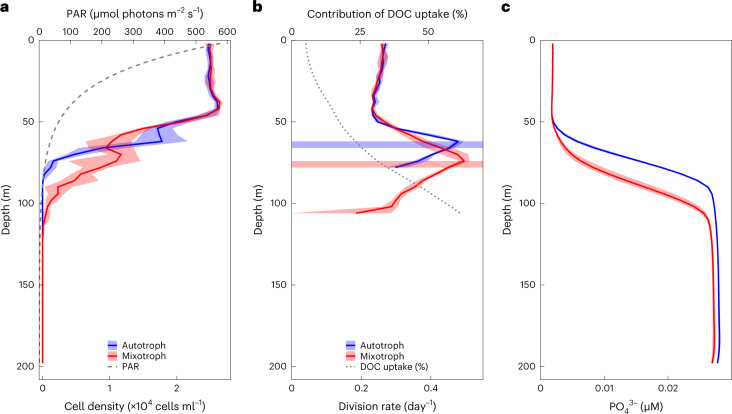
Vertical profiles of simulated autotrophs and mixotrophs in the individual-based model. The red and blue error bands in all panels indicate the minimums and maximums of an ensemble of ten model runs. **a**, Vertical profiles of cell density of simulated autotrophs (blue) and mixotrophs (red). The vertical profile of PAR is represented as the grey dashed line. **b**, Vertical profiles of cell division rate of autotrophs (blue) and mixotrophs (red). The blue and red stripes indicate the transition point from nutrient limitation to carbon limitation of phytoplankton growth. The black dotted line represents the contributions of DOC uptake to total carbon acquisition in mixotrophs. **c**, Vertical profiles of phosphate in simulations of autotroph (blue) and mixotroph (red). Source data

**Extended Data Fig. 3:**
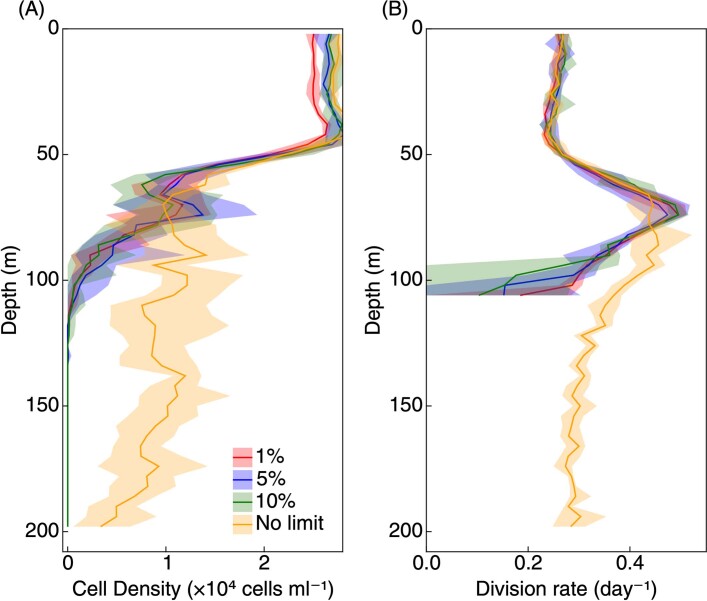
Simulations of individual-based model with different requirements of photosynthesis on the DOC uptake. Laboratory studies of *Prochlorococcus* show that they cannot survive long exposure to darkness (beyond several days) even when supplied with organic carbon sources^13,16^. In order to represent this obligate phototrophy in the model, the parameter $f_{PS}^{min}$ represents a minimum fraction of C that must originate from photosynthesis (that is Setting this parameter at 1% means the DOC uptake stops if the contribution from photosynthesis is less than 1%). To determine the sensitivity of the model results to the value of this parameter, we show different assembles of simulations with different requirements for the fraction of phototrophy (red line for 1%, blue line for 5%, green line for 10%, and yellow line for no requirement). The assemble of simulations in which DOC uptake requires at least 1% of photosynthesis contribution to total C uptake is shown in Fig. 3 of the main text. While the predicted cell density is relatively insensitive to the value of this parameter between 1–10%, without this parameter, the modeled Prochlorococcus can grow at all depths. Source data

To investigate the implications of mixotrophy on biogeochemical dynamics, we employed an individual-based modelling approach (for details, see Methods), simulating trajectories of individual *Prochlorococcus* cells (or super-agents representing many cells) through light and nutrient environments in a two-dimensional, highly resolved turbulent fluid flow (see Supplementary Video 1). Inorganic nutrients and a DOC-like tracer are represented by density-based equations. Briefly, individuals fix carbon by photosynthesis and take up inorganic nitrogen and phosphorus. Two idealized types of individuals are simulated separately, one with a strict photo-autotrophic lifestyle and the other which is mixotrophic and able to assimilate carbon from the DOC-like substance. The mixotrophic individual cannot live strictly heterotrophically, as suggested by Coe et al.^13^, which we parameterize as requiring at least 1% of the incorporated C to come from photosynthesis (Extended Data Fig. 3). In Fig. 3a, we illustrate horizontally averaged profiles of cell density from the purely autotrophic and mixotrophic simulations, illustrating how mixotrophy supports a population of *Prochlorococcus* below ~75 m. The simulated daily division rate of ~0.2 day^−1^ at depth (Fig. 3b) is consistent with the published cell-cycle profiles from the subtropics and the equatorial Pacific^31,32^ and is a bit higher than the aforementioned inferred division rate in the Mediterranean that is based on NH_4_ uptake. Mixotrophs and autotrophs share the same division rate (~0.3 day^−1^) in the mixed layer (surface 50 m) where the inorganic nutrient is the limiting factor in the simulations. The autotrophs then reach a maximum daily division rate of ~0.5 day^−1^ at 60 m depth where the transition of N to C limitation happens, and then decrease rapidly to zero at 90 m depth owing to light limitation. In contrast, the mixotrophs have a deeper maximum growth rate of ~0.5 day^−1^ at 80 m depth where the transition of N to C limitation occurs and gradually decrease to ~0.2 day^−1^ at 125 m depth (Fig. 3b). The deeper maximum division depth of the mixotrophs and their ability to maintain a population at depths where photosynthesis is not sufficient are supported by the DOC utilization, which is presented as a black line in Fig. 3b. In the mixotrophic simulation, the contribution of DOC uptake to the vertically integrated total production is ~12%, and ~43% when light becomes the limiting factor, below the red stripe in Fig. 3b. The contribution of DOC and the maximal depth at which *Prochlorococcus* can grow are broadly consistent with the division rate profile model and are sensitive to parameter values that control the nutritional value of the DOC-like substance (and that cannot be a priori constrained by empirical data at this point; Methods). Notably, the horizontal stripes in Fig. 3b indicate the depth at which limitation shifted from nutrients to C in the two ensembles of simulations. The simulated mixotrophic cells grow deeper than the purely autotrophic ones, taking up inorganic nutrients and leading to a notably deeper nutricline (Fig. 3c).

### Interpretation of vertical distributions of *Prochlorococcus* ecotypes

**Fig. 4:**
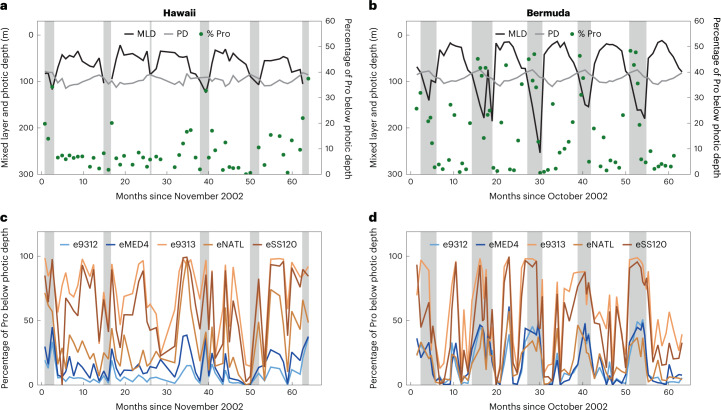
Estimating the number of *Prochlorococcus* cells and of specific ecotypes found below their photic depth at Hawaii and Bermuda. **a**,**b**, The percentage of total *Prochlorococcus* cells (Pro) found below their photic zone at Hawaii (**a**) and Bermuda (**b**), defined as the integrated illumination level supporting the growth of representative strains in laboratory cultures^24^ (grey line shows this depth for HL strains). The black line shows the mixed layer depth (MLD), the grey line shows the photic depth (PD), the green dots represent the percentages of *Prochlorococcus* (counted by flow cytometry) below the photic depth, and the grey areas represent non-stratified conditions where cells may be mixed from depth to the surface. **c**,**d**, The percentage of each *Prochlorococcus* ecotype below its photic depth, as measured by quantitative PCR. The data are taken from Malmstrom et al.^23^; for more details, see Supplementary Information. Source data

To what extent does mixotrophy support various lineages within natural, genetically diverse, populations of *Prochlorococcus*? To answer this question, we calculated the fraction of the *Prochlorococcus* cells as well as of individual ecotypes living below the depth where they can be supported by photosynthesis alone over a 5 year time series in the North Pacific and North Atlantic gyres (Hawaii and Bermuda time-series study sites, respectively^23^; for more details, see Supplementary Information). We consider only the time of year when the water column is stratified (white regions in Fig. 4), defined here as a mixed layer depth that is shallower than the photic depth (light intensity is >10 µmol photons m^−2^ s^−1^ for HL strains or >2.8 µmol photons m^−2^ s^−1^ for LL strains, experimentally determined minimal light requirement for active growth of HL- and LL-adapted strains during a 14 h:10 h day:night cycle^24^). This is because at other times cells below the photic depth but still within the upper mixed layer could be transferred closer to the surface and therefore receive increased light. An average of ~8–10% of the *Prochlorococcus* cells during these stratified periods are likely to be light starved (up to 30%, Fig. 4a,b). This includes the vast majority of cells belonging to LL-adapted ecotypes (measured using quantitative PCR^23^). LL-adapted cells encode a higher number of genes potentially involved in mixotrophy (for example, sugar and amino acid uptake^10,44^) and represent a considerable fraction of the genetic diversity of the *Prochlorococcus* ‘collective’. We propose that most of these cells require mixotrophy to survive in their deep photic zone niche (Fig. 4c,d).

## Discussion

We have presented several lines of evidence illustrating the importance of mixotrophic carbon assimilation by *Prochlorococcus*. The uptake of isotopically labelled nitrogen in samples from the Mediterranean Sea indicate doubling times at the DCM of about a week, consistent with cell-cycle-based observations from the Equatorial and Subtropical Pacific^27,29–32^. The associated uptake of labelled carbon suggests that this growth rate is viable only if more than three-quarters of assimilated carbon is sourced from organic matter. Using a laboratory-calibrated model of carbon-specific photosynthesis rates and local environmental data, we compared carbon-limited growth rates with observed cell-cycle observations at the Pacific locations. We estimated that 18–25% of depth-integrated, net carbon assimilation by *Prochlorococcus* is heterotrophic at those sites, with as much as 80% heterotrophic carbon supply at the DCM. This shifts the perception of *Prochlorococcus* as a photo-autotrophic primary producer. We do not expect products such as remote-sensing-based estimates of global-scale primary production to be strongly affected, as these are typically calibrated with data from isotopically labelled inorganic carbon studies and, hence, other sources of error notwithstanding, are appropriately estimating photosynthesis and not growth rates. We explored the wider consequences of the phenomenon in simulations with an individual-based model that resolves a DOC-like substance. These simulations suggest that such extensive mixotrophy in the deeper photic layer will notably deepen the nutricline. This is important for carbon cycle simulations, most of which do not currently resolve mixotrophy and may predict, or inappropriately tune, a too-shallow nutricline. It reminds us that ‘recyclers’ and ‘autotrophs’ may also compete for resources, affecting ecosystem dynamics^45^. Finally, investigation of the ecotypic, vertical biogeography in the subtropical North Pacific and North Atlantic^23^ indicates that LL-adapted *Prochlorococcus* spend 50–100% of their time, depending on season, below the deepest horizon for photo-autotrophically viable maintenance of the population. We propose that reliance on mixotrophy, rather than on photosynthesis, underpins the ecological success of a large fraction of the global *Prochlorococcus* population and its collective genetic diversity.

## Methods

### Isotope labelling and phylogenetic analysis of a natural marine bacterioplankton population at sea

Mediterranean seawater was collected during August 2017 (station N1200, 32.45° N, 34.37 °E) from 11 depths by Niskin bottles and divided into triplicate 250 ml polycarbonate bottles. Two bottles from each depth were labelled with 1 mM sodium bicarbonate-^13^C and 1 mM ammonium-^15^N chloride (Sigma-Aldrich), and all three bottles (two labelled and one control) were incubated at the original depth and station at sea for 3.5 h around mid-day. The stable isotopes were chosen to enable direct comparison of C and N uptake in single cells, and the short incubation time was chosen to minimize isotope dilution and potential recycling and transfer of ^13^C and ^15^N between community members^25^. After incubation, bottles were brought back on board and the incubations were stopped by fixing with 2× electron-microscopy-grade glutaraldehyde (2.5% final concentration) and stored at 4 °C until sorting analysis. Cell sorting, NanoSIMS analyses and the calculation of uptake rates were performed as described in Roth-Rosenberg et al.^26^.

### DNA collection and extraction from seawater

Samples for DNA were collected on 0.22 µm Sterivex filters (Millipore). Excess water was removed using a syringe, 1 ml lysis buffer (40 mM EDTA, 50 mM Tris pH 8.3, and 0.75 M sucrose) was added and both ends of the filter were closed with parafilm. Samples were kept at −80 °C until extraction. DNA was extracted by using a semi-automated protocol including manual chemical cell lysis before automated steps using the QIAamp DNA Mini Protocol: DNA Purification from Blood or Body Fluids (Spin Protocol, starting from step 6, at the BioRap unit, Faculty of Medicine, Technion). The manual protocol began with thawing the samples, then the storage buffer was removed using a syringe and 170 µl lysis buffer added to the filters. Thirty microlitres of Lysozyme (20 mg ml^−1^) were added to the filters and incubated at 37 °C for 30 min. After incubation, 20 µl proteinase K and 200 µl buffer AL (from the Qiagen kit) were added to the tube for 1 h at 56 °C (with agitation). The supernatant was transferred to a new tube, and DNA was extracted using the QIAcube automated system. All DNA samples were eluted in 100 μl DNA-free distilled water.

### ITS PCR amplification

PCR amplification of the ITS was carried out with specific primers for *Prochlorococcus* CS1_16S_1247F (5′-ACACTGACGACATGGTTCTACACGTACTACAATGCTACGG) and Cs2_ITS_Ar (5′-TACGGTAGCAGAGACTTGGTCTGGACCTCACCCTTATCAGGG)^21,22^. The first PCR was performed in triplicate in a total volume of 25 μl containing 0.5 ng of template, 12.5 μl of MyTaq Red Mix (Bioline) and 0.5 μl of 10 μM of each primer. The amplification conditions comprised steps at 95 °C for 5 min, 28/25 (16 S/ITS) cycles at 95 °C for 30 s, 50 °C for 30 s and 72 °C for 1 min followed by one step of 5 min at 72 °C. All PCR products were validated on a 1% agarose gel, and triplicates were pooled. Subsequently, a second PCR amplification was performed to prepare libraries. These were pooled and after a quality control sequenced (2 × 250 paired-end reads) using an Illumina MiSeq sequencer. Library preparation and pooling were performed at the DNA Services facility, Research Resources Center, University of Illinois at Chicago. MiSeq sequencing was performed at the W.M. Keck Center for Comparative and Functional Genomics at the University of Illinois at Urbana-Champaign.

### ITS sequence processing

Paired-end reads were analysed using the Dada2 pipeline^46^. The quality of the sequences per sample was examined using the Dada2 ‘plotQualityProfile’ command. Quality filtering was performed using the Dada2 ‘filterAndTrim’ command with parameters for quality filtering truncLen=c(290,260), maxN=0, maxEE=c(2,2), truncQ=2, rm.phix=TRUE, trimLeft=c(20,20). Following error estimation and dereplication, the Dada2 algorithm was used to correct sequences. Merging of the forward and reverse reads was done with minimum overlap of 4 bp. Detection and removal of suspected chimaeras was done with command ‘removeBimeraDenovo’. In total, 388,417 sequences in 484 amplicon sequence variants were counted. The amplicon sequence variants were aligned in MEGA6 (ref. ^47^), and the first ~295 nucleotides, corresponding to the 16S gene, were trimmed. The ITS sequences were then classified using BLASTn against a custom database of ITS sequences from cultured *Prochlorococcus* and *Synechococcus* strains as well as from uncultured HL and LL clades.

### Individual-based model

PlanktonIndividuals.jl (v0.1.9) was used to run the individual-based simulations^48^. Briefly, the cells fix inorganic carbon through photosynthesis and nitrogen, phosphorus and DOC from the water column into intracellular quotas and grow until division or grazing. Cell division is modelled as a probabilistic function of cell size. Grazing is represented by a quadratic probabilistic function of cell population. Cells consume nutrient resources, which are represented as Eulerian, density-based tracers. A full documentation of state variables and model equations are available online at https://juliaocean.github.io/PlanktonIndividuals.jl/dev/. Equations related to mixotrophy are shown below as an addition to the online documentation.

1 $$V_{{\mathrm{DOC}}} = V_{{\mathrm{DOC}}}^{{\mathrm{max}}} \cdot {{\mathrm{max}}}\left( {0.0,{{\mathrm{min}}}\left( {1.0,\,\frac{{q_{\mathrm{C}}^{{\mathrm{max}}} - q_{\mathrm{C}}}}{{q_{\mathrm{C}}^{{\mathrm{max}}} - q_{\mathrm{C}}^{{\mathrm{min}}}}}} \right)} \right) \cdot \frac{{{\mathrm{DOC}}}}{{{\mathrm{DOC}} + K_{{\mathrm{DOC}}}^{{\mathrm{sat}}}}}$$

2 $$f_{{\mathrm{PS}}} = \frac{{P_{\mathrm{S}}}}{{P_{\mathrm{S}} + V_{{\mathrm{DOC}}}}}$$

3 $$V_{{\mathrm{DOC}}} = 0,\,{\mathrm{if}}\,f_{{\mathrm{PS}}} < f_{{\mathrm{PS}}}^{{\mathrm{min}}}$$

where *V*_*DOC*_ is the cell-specific DOC uptake rate (mol C cell^−1^ s^−1^), $V_{{\mathrm{DOC}}}^{{\mathrm{max}}}$ is the maximum cell-specific DOC uptake rate (mol C cell^−1^ s^−1^), $q_{\mathrm{C}}^{{\mathrm{max}}}$ is the maximum cell carbon quota (mol C cell^−1^), $q_{\mathrm{C}}^{{\mathrm{min}}}$ is the minimum cell carbon quota (mol C cell^−1^). The maximum and minimum functions here is used to keep *q*_C_ between $q_{\mathrm{C}}^{{\mathrm{min}}}$ and $q_{\mathrm{C}}^{{\mathrm{max}}}$. $K_{{\mathrm{DOC}}}^{{\mathrm{sat}}}$ is the half-saturation constant for DOC uptake (mol C m^−3^). *f*_PS_ is the fraction of fixed C originating from photosynthesis (*P*_S_, mol C cell^−1^ s^−1^). DOC uptake stops when *f*_PS_ is smaller than $f_{{\mathrm{PS}}}^{{\mathrm{min}}}$(minimum fraction of fixed C originating form photosynthesis, 1% by default) according to laboratory studies of *Prochlorococcus* that showed that they cannot survive long exposure to darkness (beyond several days) even when supplied with organic carbon sources^13^. (1 − *f*_PS_) is also shown in Fig. 3 as the contribution of DOC uptake.

We set up two separate simulations; each of them has a population of either an obligate photo-autotroph or a mixotroph that also consumes DOC. The initial conditions and parameters (Supplementary Table 3) are the same for the two simulations except the ability of mixotrophy. The simulations were run with a timestep of 1 min for 360 simulated days to achieve a steady state. We run the two simulations for multiple times in order to get the range of the stochastic processes.

### Evaluation of autotrophic growth rates

We evaluated the carbon-specific, daily-averaged carbon fixation rate, ℙ as a function of light intensity (*I*, µE), following Platt et al.^33^:

4 $${\Bbb P} = \frac{1}{{\Delta t}}{\int}_0^{\Delta t} {\frac{{q_{{\mathrm{Chl}}}}}{{q_{\mathrm{C}}}}} P_{\mathrm{S}}^{{\mathrm{Chl}}}\left( {1 - e^{ - \alpha _{{\mathrm{Chl}}}I/P_{\mathrm{S}}^{{\mathrm{Chl}}}}} \right)e^{ - \beta _{{\mathrm{Chl}}}I/P_{\mathrm{S}}^{{\mathrm{Chl}}}}\Delta t$$

Here, $P_{\mathrm{S}}^{{\mathrm{Chl}}}$, *α*_Chl_ and *β*_Chl_ are empirically determined coefficients representing the chlorophyll-*a*-specific carbon fixation rate (mol C (mol Chl)^−1^ s^−1^), the initial slope of the photosynthesis–light relationship and photo-inhibition effects at high photon fluxes, respectively. We impose empirically determined values for $P_{\mathrm{S}}^{{\mathrm{Chl}}}$, *α*_Chl_ and *β*_Chl_ from the published study of Moore and Chisholm^24^. The natural *Prochlorococcus* community comprises HL and LL ecotypes, which have different values of $P_{\mathrm{S}}^{{\mathrm{Chl}}}$, *α*_Chl_ and *β*_Chl_, and the community growth rate is expected to be between that of HL extremes and LL extremes. Therefore, we use photo-physiological parameters for an HL-adapted ecotype (MIT9215), acclimated at 70 µmol photons m^−2^ s^−1^ and an LL-adapted ecotype (MIT9211), acclimated 9 µmol photons m^−2^ s^−1^. The models with these values are shown as the different lines in Fig. 2b,d. *I* is the hourly PAR, estimated by scaling the observed noon value at each depth with a diurnal variation evaluated from astronomical formulae based on geographic location and time of year^37,38^.

$\frac{{q_{{\mathrm{Chl}}}}}{{q_{\mathrm{C}}}}$ is the molar chlorophyll-*a* to carbon ratio, which is modelled as a function of growth rate and light intensity using the Inomura^34^ model (equation 17 therein) where parameters were calibrated with laboratory data from Healey^49^. In addition, the maximum growth rate ($\mu _{{\mathrm{max}}}^I$) based on macromolecular allocation is also estimated using the Inomura model (equation 30 therein). An initial guess of the growth rate and the empirically informed light intensity are used to estimate $\frac{{q_{{\mathrm{Chl}}}}}{{q_{\mathrm{C}}}}$, which is then used to evaluate the light-limited, photoautotrophic growth rate

5 $${\Bbb V}_{\mathrm{C}}^{{\mathrm{auto}}} = \min \left( {{\Bbb P} - K_{\mathrm{R}},\mu _{{\mathrm{max}}}^I} \right)$$

from which the $\frac{{q_{{\mathrm{Chl}}}}}{{q_{\mathrm{C}}}}$ is again updated. The light-limited growth rate is used to re-evaluate the $\frac{{q_{{\mathrm{Chl}}}}}{{q_{\mathrm{C}}}}$. Repeating this sequence until the values converge, ${\Bbb V}_{\mathrm{C}}^{{\mathrm{auto}}}$ and $\frac{{q_{{\mathrm{Chl}}}}}{{q_{\mathrm{C}}}}$ are solved iteratively.

The nitrogen-specific uptake rate of fixed nitrogen (day^−1^) is modelled as

6 $${\Bbb V}_{{{\mathrm{N}}}} = {\Bbb V}_{\mathrm{N}}^{{\mathrm{max}}}\frac{1}{{Q_{\mathrm{N}}}}\frac{N}{{N + K_{{{\mathrm{N}}}}}}$$

where values of the maximum uptake rate, ${\Bbb V}_{\mathrm{N}}^{{\mathrm{max}}}$, and half-saturation, *K*_N_, are determined from empirical allometric scalings^35^, along with a nitrogen cell quota *Q*_N_ from Bertilsson et al.^39^.

The P-limited growth rate, or the phosphorus-specific uptake rate of phosphate (day^−1^), is modelled as

7 $${\Bbb V}_{\mathrm{P}} = {\Bbb V}_{\mathrm{P}}^{{\mathrm{max}}}\frac{1}{{Q_{\mathrm{P}}}}\frac{{{\mathrm{PO}_{4}}^{3 - }}}{{{\mathrm{PO}_{4}}^{3 - } + K_{\mathrm{P}}}}$$

where values of the maximum uptake rate, ${\Bbb V}_{\mathrm{P}}^{{\mathrm{max}}}$. and half-saturation, *K*_P_, are determined from empirical allometric scalings^35^, along with a nitrogen cell quota *Q*_P_ from Bertilsson et al.^39^.

Iron uptake is modelled as a linear function of cell surface area (SA), with rate constant ($k_{{\mathrm{Fe}}}^{{\mathrm{SA}}}$) following Lis et al.^36^.

8 $${\Bbb V}_{{\mathrm{Fe}}} = k_{{\mathrm{Fe}}}^{{\mathrm{SA}}} \cdot {\mathrm{SA}}\frac{1}{{Q_{{\mathrm{Fe}}}}}{\mathrm{Fe}}$$

The potential light-, nitrogen-, phosphorus- and iron-limited growth rates (${\Bbb V}_{\mathrm{C}},{\Bbb V}_{\mathrm{N}},{\Bbb V}_{\mathrm{P}},{\Bbb V}_{{\mathrm{Fe}}}$) were evaluated at each depth in the water column and the minimum is the local modelled photo-autotrophic growth rate estimate, assuming no mixotrophy (Fig. 2b,d, blue lines). Parameters used in this evaluation are listed in Supplementary Table 2.

An important premise of this study is that heterotrophy is providing for the shortfall in carbon under very low light conditions, but not nitrogen. It is known that *Prochlorococcus* can assimilate amino acids^9^ and therefore the stoichiometry of the heterotrophic contribution might alter the interpretations. However, it is also known that *Prochlorococcus* can exude amino acids^40^, which might cancel out the effects on the stoichiometry of *Prochlorococcus*.

For the estimates of phototrophic growth rate from local environmental conditions (Fig. 2) we employed photo-physiological parameters from laboratory cultures of *Prochlorococcus*^24^. For the purposes of this study, we have assumed that the photosynthetic rates predicted are net primary production, which means that autotrophic respiration has been accounted for in the measurement. However, the incubations in that study were of relatively short timescale (45 min), which might suggest they are perhaps more representative of gross primary production. If this is the case, our estimates of photo-autotrophic would be even lower after accounting for autotrophic respiration, and thus would demand a higher contribution from heterotrophic carbon uptake. In this regard, our estimates might be considered a lower bound for organic carbon assimilation.

### Reporting summary

Further information on research design is available in the Nature Research Reporting Summary linked to this article.

## Supplementary information

Supplementary Information Supplementary Text 1 and 2, Tables 1–3 and Fig. 1.

Reporting Summary

Supplementary Video 1 Simulation of the individual-based model.

## Figures and Tables

**Extended Data Fig. 1:**
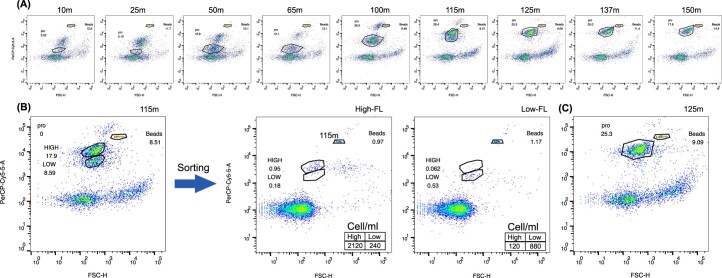
Gating and sorting of Prochlorococcus cells from the Eastern Mediterranean. (**a**) Depth profile of flow cytometry scatterplots, with the gating used to count *Prochlorococcus* populations shown. The x axis is forward scatter (height) from 10°-10^5^ arbitrary units, the Y axis is chlorophyll autofluorescence (PerCP-Cy5, Ex=488, Em=680) with arbitrary units from 10^1^-10^5^. The standard fluorescent beads are also shown. The cell counts from this analysis are presented in Fig. 1b, and the fluorescence histograms in Fig. 1d. (**b**), (**c**) Sorting gates for ^13^C- and ^15^N-labeled *Prochlorococcus* cells prior to nanoSIMS analysis. In panel B the purity of the sorted fractions is shown. No purity analysis was performed for the 125 m sample, as the goal was not to differentiate between co-occurring sub-populations. Source data

## Data Availability

The oceanographic measurements (CTD data, nutrient concentrations and cell counts) have been submitted to the BCO-DMO database under acronym HADFBA (dataset name ‘EMS photic zone’). Raw ITS sequences are available from GenBank under BioProject PRJNA802335, accessions SAMN25553516-SAMN25553524. Source data are provided with this paper.

## Source data

Source Data Fig. 1 Statistical source data.
Source Data Fig. 2 Statistical source data.
Source Data Fig. 3 Statistical source data.
Source Data Fig. 4 Statistical source data.
Source Data Extended Data Fig. 1 Statistical source data.
Source Data Extended Data Fig. 2 Statistical source data.
Source Data Extended Data Fig. 3 Statistical source data.

## Code availability

The R package Dada2 (v1.16) is used to process ITS sequence. BLASTn was used to analyse the ITS sequences and FloJo 10.5.3 to analyse the flow cytometer data. The codes to estimate autotrophic growth rates and to run individual-based simulations are available at Github (https://github.com/zhenwu0728/Prochlorococcus_Mixotrophy) with detailed instructions under MIT license.
