# Supplementary material for: Single-cell measurements and modelling reveal substantial organic carbon acquisition by Prochlorococcus
Source: Nat Microbiol. 2022 Nov 3;7(12):2068–77. doi: 10.1038/s41564-022-01250-5 (PMC9712107; doi:10.1038/s41564-022-01250-5)
Supplement: Supplementary file 1 — Supplementary Text 1 and 2, Tables 1–3 and Fig. 1. [file 41564_2022_1250_MOESM1_ESM.pdf]

nature microbiology

Article https://doi.org/10.1038/s41564-022-01250-5

Single-cell measurements and modelling reveal substantial organic carbon acquisition by *Prochlorococcus*

In the format provided by the authors and unedited

Single cell measurements and modelling reveal significant organic carbon acquisition by *Prochlorococcus*

Zhen Wu[1]#, Dikla Aharonovich[2]#, Dalit Roth-Rosenberg[2], Osnat Weissberg[2], Tal Luzzatto-Knaan[2], Angela Vogts[3], Luca Zoccarato[4], Falk Eigemann[3], Hans-Peter Grossart[4,5], Maren Voss[3], Michael J. Follows*[1] and Daniel Sher *[2]

[1]Department of Earth, Atmospheric and Planetary Sciences, Massachusetts Institute of Technology, Cambridge, MA 02139, USA

[2]Department of Marine Biology, Leon H. Charney School of Marine Sciences, University of Haifa, 31905, Israel

[3]Leibniz-Institute for Baltic Sea Research, Seestrasse 15, D-18119 Warnemuende, Germany

[4]Department of Experimental Limnology, Leibniz-Institute of Freshwater Ecology and Inland Fisheries, Alte Fischerhuette 2, D-16775 Stechlin, Germany

[5]Potsdam University, Institute of Biochemistry and Biology, Maulbeeralle 2, D-14469 Potsdam, Germany

* Corresponding authors: mick@ocean.mit.edu, dsher@univ.haifa.ac.il

# These authors contributed equally to this study

Supplementary text S1 to S2

Supplementary table S1 to S3

Supplementary figure S1

Text S1 Estimating the fraction of *Prochlorococcus* cells living mixotrophically

A rough estimate of the fraction of *Prochlorococcus* cells living mixotrophically can be obtained by determining how many cells are found under conditions where the light intensity is not sufficient to support active growth.

We first estimated the minimal amount of light (integrated over a 24-hour period) required for *Prochlorococcus* cells from different ecotypes to grow under lab conditions, using the measurements of Moore and Chisholm[1]. The minimal light levels that supported the growth of HL and LL strains were 10 μmol photons m^{-2} s^{-1} and 2.8 μmol photons m^{-2} s^{-1} for HL and LL strains, respectively, during a 14:10 day-night cycle. We converted these to integrated daily PAR levels (in mole photons m^{-2} s^{-1}). The eMIT9313 ecotype is treated as a LL strain in this analysis despite the limited light levels analyzed for the MIT9313 strain in the Moore and Chisholm study[1]. The threshold light levels based on this calculation were 0.50 mol photons m^{-2} day^{-1} for HL strains and 0.18 mol photons m^{-2} day^{-1} for LL strains.

We then determined the number of *Prochlorococcus* cells (total *Prochlorococcus* population and individual ecotypes) found at depths where the integrated daily PAR is lower than the minimal light requirement (i.e. below the photic depth for *Prochlorococcus*). For this analysis, we used the dataset generated by Malmstrom and colleagues over 5-year time series at the Hawaii and Bermuda time series study sites (HOT and BATS[2]). This dataset includes estimates of light penetration to different depths, counts of total *Prochlorococcus* cells obtained using flow cytometry, and quantitative PCR estimates of the cell abundance of each ecotype. We defined the *Prochlorococcus* photic depth as the depth where light intensity is above the threshold levels described above (grey line in Figure 1A, B represents this threshold for HL cells). We then calculated the integrated cell numbers (either flow cytometry counts or qPCR-based counts of individual ecotypes) found below this depth and divided this number by the total integrated cell counts to obtain the fraction of cells living below the threshold light level. This could be performed for all cruises apart from HOT161, 169, 186 and 192, for which qPCR counts were not available.

In our analysis we consider only the time of the year when the water column is stratified (defined here as a mixed layer depth that is shallower than the HL *Prochlorococcus* photic depth), because at other times cells below the photic depth but still within the upper mixed layer could be mixed closer to the surface and therefore receive sufficient light. During these times, the estimated photic depth for HL ecotypes was between 83-114m at HOT and 81-110m at BATS, and for LL ecotypes between 101-140m at HOT and 103-135m at BATS. An average of ~8-10% of the *Prochlorococcus* cells at HOT and BATS, respectively, are found below these depths (range ~1.4-37.5% at HOT and ~0.5-43% at BATS).

Finally, we estimated, in the same way, the fraction of cells (based on qPCR measurements) from each ecotype found below the photic depth defined for HL or LL ecotypes (Figure 4C, D, Table S1). These results show that while an average of 6-14% of the HL ecotypes live below their photic zone, a much larger percent of the LL-II/III (eSS120) and LL-IV (e9313) ecotypes live deeper than supported by phototrophy alone (35-72%). As expected, the LL-I (eNATL) showed an intermediate pattern.

We note several caveats associated with this analysis. Firstly, the counts of total *Prochlorococcus* cells and of individual ecotypes were performed using different methods, and the qPCR counts do not sum up to 100% of the total cells (discussed in the supporting information of Malmstrom et al[2]). This suggests that some part of the *Prochlorococcus* diversity is missing from the genetic analysis, and indeed it is now known that there are additional ecotypes beyond those measured by Malmstrom and colleagues (e.g., Berube et al.[3] and Biller et al.[4]). Secondly, there are oceanic processes such as internal waves that are not included in our calculation but may affect the depth distribution of *Prochlorococcus* cells, and thus their history of light exposure. Finally, metabolic modeling suggests that *Prochlorococcus* strains may be able to grow at light levels below the minimal light flux supporting growth in laboratory cultures[5]. Future studies are needed in order to better constrain these rough estimates of the percent of mixotrophic *Prochlorococcus*, incorporating better estimates of the minimum light needed for growth, taking into account processes leading to mixing of cells from below the mixed layer (e.g., internal waves) and also accounting for other *Prochlorococcus* ecotypes not analyzed here.

Text S2 Evaluation of cell counts of *Prochlorococcus* in the Pacific Ocean

We built a one-dimensional steady state model of carbon-based phytoplankton biomass (B_C, $mol\ C\ m^{-3}$) based on the equations used to evaluate the autotrophic growth rates. For simplicity, here we assume that cellular carbon quota (Q_C) is fixed, and cell density ($\mathbb{X}$, $cells\ m^{-3}$) is B_C/Q_C.

$$\frac{dB_C}{dt} = \min(\mathbb{P}, \mathbb{V}_N, \mathbb{V}_P, \mathbb{V}_{Fe})\, B_C - \ K_R B_C - mB_C^2 - \nabla \cdot (\vec{u} B_C) \tag{1}$$

where $\mathbb{P}$ is the carbon-specific rate of net primary production (day^{-1}), $\mathbb{V}_N$, $\mathbb{V}_P$, and $\mathbb{V}_{Fe}$ are nutrient-specific nutrient uptake rate (day^{-1}, nitrogen, phosphorus, and iron respectively). Mortality (m) is assumed to have a quadratic dependence on the biomass and rate coefficient ($(mol\ C\ m^{-3})^{-1} \cdot day^{-1}$) which is reasonable if the biomass of the predator population is in equilibrium with that of the prey[6]. K_R is a fixed-rate of maintenance respiration (day^{-1}). $\nabla \cdot (\vec{u} B_C)$ represents the physical transport of the organisms by turbulent flow which, for the purposes of this simplified model, is assumed to be negligible. In other words, we assume that the biomass concentration is determined largely by the local ecological dynamics and not by physical transport.

At steady state, $\frac{dB_C}{dt} = 0$, Eq.1 is solved for B_C:

$$B_C = \frac{\min(\mathbb{P}, \mathbb{V}_N, \mathbb{V}_P, \mathbb{V}_{Fe}) - \ K_R}{m} \tag{2}$$

$\min(\mathbb{P}, \mathbb{V}_N, \mathbb{V}_P, \mathbb{V}_{Fe}) - \ K_R$ is the growth rate (μ) evaluated in the manuscript. Eq.2 is finally:

$$B_C = \ \mu/m \tag{3}$$

$$\mathbb{X} = \ \mu/mQ_c \tag{4}$$

The evaluations of cell counts are shown in Fig.S4.

Table S1 - mean, standard deviation and maximum percent of each *Prochlorococcus* ecotype found below the photic depth at HOT and BATS.

	HOT mean ± SD	HOT maximum	BATS mean ± SD	BATS maximum
**e9312**	6.85 ± 6.58	36.67	8.64 ± 11.16	42.70
**eMED4**	14.54 ± 10.08	38.72	10.23 ± 14.04	60.98
**eNATL**	31.39 ± 22.26	97.38	12.22 ± 12.59	57.27
**eSS120**	60.45 ± 27.90	99.25	35.91 ± 30.20	99.71
**e9313**	72.43 ± 24.28	98.12	47.50 ± 29.73	99.32

Table S2. Parameters used in the evaluation of autotrophic growth rates in Pacific Ocean.

Symbol	Description	High-light	Low-light	Unit	Reference
$\boldsymbol{P_S^{Chl}}$	Chlorophyll-a specific carbon fixation rate	8.0	3.0	$fg\ C \cdot (fg\ Chl)^{-1} \cdot hour^{-1}$	Moore and Chisholm[1]
$\boldsymbol{\alpha_{Chl}}$	Initial slope of the photosynthesis-light curve	0.05	0.09		Moore and Chisholm[1]
$\boldsymbol{\beta_{Chl}}$	Photo-inhibition effects	0.008	0.010		Moore and Chisholm[1]
$\mathbb{V}_{\boldsymbol{N}}^{\boldsymbol{max}}$	Maximum nitrogen uptake rate	1.94×10^{-9}		$\mu mol\ N \cdot day^{-1} \cdot cell^{-1}$	Edwards et al.[7]
$\boldsymbol{K_N}$	Half-saturation constant of nitrogen uptake	8.20×10^{-2}		$\mu mol\ N \cdot L^{-1}$	Edwards et al.[7]
$\mathbb{V}_{\boldsymbol{P}}^{\boldsymbol{max}}$	Maximum phosphorus uptake rate	1.43×10^{-9}		$\mu mol\ P \cdot day^{-1} \cdot cell^{-1}$	Edwards et al.[7]
$\boldsymbol{K_P}$	Half-saturation constant of phosphorus uptake	1.97×10^{-2}		$\mu mol\ P \cdot L^{-1}$	Edwards et al.[7]
$\boldsymbol{K_{Fe}^{SA}}$	Iron surface area-specific uptake constant	1.0×10^{-9}		$L \cdot day^{-1} \cdot \mu m^{-2}$	Lis et al.[8]
$\boldsymbol{SA}$	Cell surface area	1.54		$\mu m^2 \cdot cell^{-1}$	
$\boldsymbol{Q_C}$	Carbon cell quota	5.08		$fmol\ C \cdot cell^{-1}$	Bertilsson et al.[9]
$\boldsymbol{Q_N}$	Nitrogen cell quota	0.77		$fmol\ N \cdot cell^{-1}$	Bertilsson et al.[9]
$\boldsymbol{Q_P}$	Phosphorus cell quota	0.048		$fmol\ P \cdot cell^{-1}$	Bertilsson et al.[9]
$\boldsymbol{Q_{Fe}}$	Iron cell quota	8.12×10^{-5}		$fmol\ Fe \cdot cell^{-1}$	Hawco et al.[10]
$\boldsymbol{K_R}$	Maintenance respiration rate	0.05		day^{-1}	Marañón et al.[11]

Table S3. Parameters used in the individual-based model.

Symbol	Description	Value	Unit
P_S^{Chl}	Cell-specific photosynthesis rate	7.14×10^{-17}	$mmol\ C \cdot cell^{-1} \cdot s^{-1}$
α_{Chl}	Irradiance absorption coefficient	2.0×10^{-2}	$m^2 \cdot mgChl^{-1}$
Φ	Maximum quantum yield	4.0×10^{-5}	$mmol\ C \cdot \mu mol\ photons^{-1}$
V_{NH4}^{max}	Maximum ammonium uptake rate	1.39×10^{-17}	$mmol\ N \cdot cell^{-1} \cdot s^{-1}$
K_{NH4}^{sat}	Half-saturation constant of ammonium uptake	0.05	$mmol\ N \cdot L^{-1}$
V_{NO3}^{max}	Maximum nitrate uptake rate	1.39×10^{-17}	$mmol\ N \cdot cell^{-1} \cdot s^{-1}$
K_{NO3}^{sat}	Half-saturation constant of nitrate uptake	0.10	$mmol\ N \cdot L^{-1}$
V_{PO4}^{max}	Maximum phosphate uptake rate	1.39×10^{-18}	$mmol\ P \cdot cell^{-1} \cdot s^{-1}$
K_{PO4}^{sat}	Half-saturation constant of phosphate uptake	0.03	$mmol\ P \cdot L^{-1}$
q_N^{max}	Maximum nitrogen cell quota	0.25	$mmol\ N \cdot mmol\ C^{-1}$
q_N^{min}	Minimum nitrogen cell quota	0.10	$mmol\ N \cdot mmol\ C^{-1}$
q_P^{max}	Maximum phosphorus cell quota	0.019	$mmol\ P \cdot mmol\ C^{-1}$
q_P^{min}	Minimum phosphorus cell quota	0.006	$mmol\ P \cdot mmol\ C^{-1}$
K_R	Maintenance respiration rate	2.31×10^{-19}	$mmol\ C \cdot cell^{-1} \cdot s^{-1}$
R_{NC}	N:C ratio in function biomass	0.15	$mmol\ N \cdot mmol\ C^{-1}$
R_{PC}	P:C ratio in function biomass	9.4×10^{-3}	$mmol\ P \cdot mmol\ C^{-1}$
V_{DOC}^{max}	Maximum DOC uptake rate (Mixotrophy only)	9.26×10^{-18}	$mmol\ C \cdot cell^{-1} \cdot s^{-1}$
K_{DOC}^{sat}	Half-saturation constant of DOC uptake (Mixotrophy only)	0.15	$mmol\ C \cdot L^{-1}$
q_C^{max}	Maximum cell quota of carbon storage (Mixotrophy only)	0.40	$mmol\ C \cdot mmol\ C^{-1}$
q_C^{min}	Minimum cell quota of carbon storage (Mixotrophy only)	0.10	$mmol\ C \cdot mmol\ C^{-1}$
f_{PS}^{min}	Minimum fraction of C that originating from photosynthesis(Mixotrophy only)	0.01	

Figure S1. Sensitivity analysis for the evaluation of autotrophic growth rate. The photosynthesis parameterization of High-Light ecotype without photo-inhibition is chosen as the base scenario. *Prochlorococcus* is nitrogen limited at Station ALOHA and iron limited at Equatorial Pacific, so maximum nitrogen uptake rate (VN at HOT, panel A) and half saturation constant of nitrogen (KN at HOT, panel B), and iron uptake constant (KFe at EqPac, panel C) are chosen for the sensitivity analysis. ±50% of variation is applied to each parameter which is indicated by blue dashed lines. Solid blue lines represent the base scenarios and red lines with dots and error bars represent the observations.

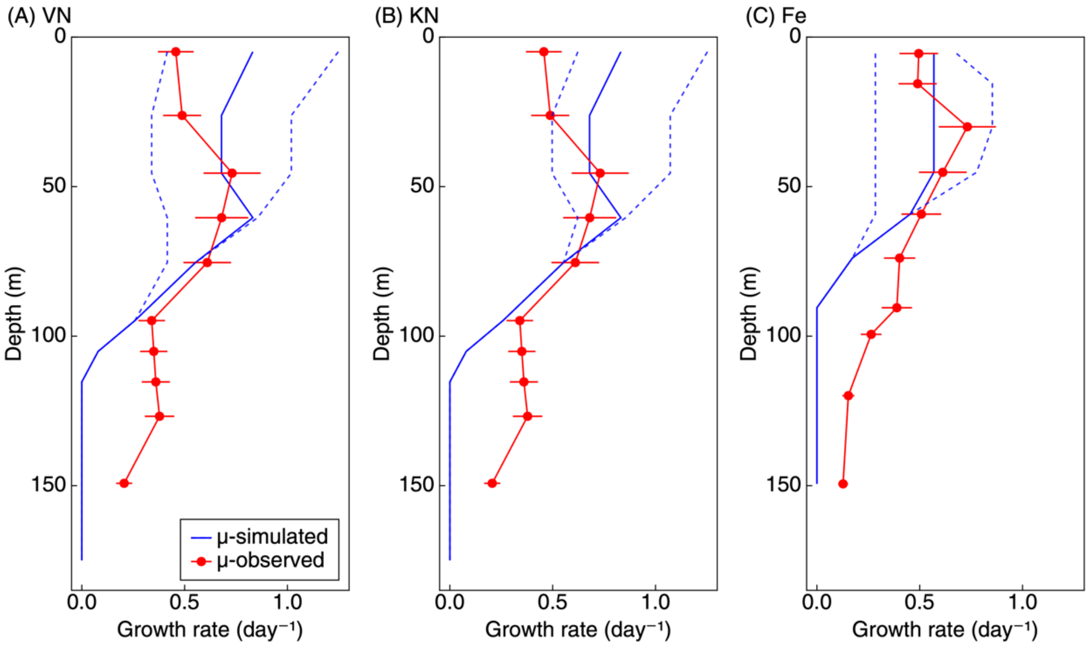

References

1. Moore, L.R. and S.W. Chisholm, Photophysiology of the marine cyanobacterium Prochlorococcus: Ecotypic differences among cultured isolates. Limnology and Oceanography, 1999. 44(3): p. 628-638.
2. Malmstrom, R.R., et al., Temporal dynamics of Prochlorococcus ecotypes in the Atlantic and Pacific oceans. Isme J, 2010.
3. Berube, P.M., et al., Single cell genomes of Prochlorococcus, Synechococcus, and sympatric microbes from diverse marine environments. Scientific data, 2018. 5: p. 180154-180154.
4. Biller, S.J., et al., Prochlorococcus: the structure and function of collective diversity. Nature Reviews Microbiology, 2014. 13: p. 13.
5. Casey, J.R., et al., Basin-scale biogeography of marine phytoplankton reflects cellular-scale optimization of metabolism and physiology. Science Advances, 2022. 8(3): p. eabl4930.
6. Gentleman, W., et al., Functional responses for zooplankton feeding on multiple resources: a review of assumptions and biological dynamics. Deep Sea Research Part II, 2003. 50: p. 2847-2875.
7. Edwards, K. F., Thomas, M. K., Klausmeier, C. A. & Litchman, E. Allometric scaling and taxonomic variation in nutrient utilization traits and maximum growth rate of phytoplankton. Limnology and Oceanography 57, 554–566 (2012).
8. Lis, H., Shaked, Y., Kranzler, C., Keren, N. & Morel, F. M. M. Iron bioavailability to phytoplankton: an empirical approach. The ISME Journal 2015 9:4 9, 1003–1013 (2014).
9. Bertilsson, S., Berglund, O., Karl, D. M. & Chisholm, S. W. Elemental composition of marine Prochlorococcus and Synechococcus: Implications for the ecological stoichiometry of the sea. Limnology and Oceanography 48, 1721–1731 (2003).
10. Hawco, N. J., Fu, F., Yang, N., Hutchins, D. A. & John, S. G. Independent iron and light limitation in a low-light-adapted Prochlorococcus from the deep chlorophyll maximum. The ISME Journal 2020 15:1 15, 359–362 (2020).
11. Marañón, E. et al. Unimodal size scaling of phytoplankton growth and the size dependence of nutrient uptake and use. Ecology Letters 16, 371–379 (2013).
