# Supplementary material for: Single-cell measurements and modelling reveal substantial organic carbon acquisition by Prochlorococcus
Source: Nat Microbiol. 2022 Nov 3;7(12):2068–77. doi: 10.1038/s41564-022-01250-5 (PMC9712107; doi:10.1038/s41564-022-01250-5)
Supplement: Supplementary file 2 — Reporting Summary [file 41564_2022_1250_MOESM2_ESM.pdf]

nature portfolio

Corresponding author(s): Mick Follows, Daniel Sher

Last updated by author(s): 2022/08/17

Reporting Summary

Nature Portfolio wishes to improve the reproducibility of the work that we publish. This form provides structure for consistency and transparency in reporting. For further information on Nature Portfolio policies, see our Editorial Policies and the Editorial Policy Checklist.

Statistics

For all statistical analyses, confirm that the following items are present in the figure legend, table legend, main text, or Methods section.

n/a	Confirmed	
☐	☒	The exact sample size (n) for each experimental group/condition, given as a discrete number and unit of measurement
☐	☒	A statement on whether measurements were taken from distinct samples or whether the same sample was measured repeatedly
☒	☐	The statistical test(s) used AND whether they are one- or two-sided<br>*Only common tests should be described solely by name; describe more complex techniques in the Methods section.*
☒	☐	A description of all covariates tested
☒	☐	A description of any assumptions or corrections, such as tests of normality and adjustment for multiple comparisons
☐	☒	A full description of the statistical parameters including central tendency (e.g. means) or other basic estimates (e.g. regression coefficient) AND variation (e.g. standard deviation) or associated estimates of uncertainty (e.g. confidence intervals)
☒	☐	For null hypothesis testing, the test statistic (e.g. F, t, r) with confidence intervals, effect sizes, degrees of freedom and P value noted<br>*Give P values as exact values whenever suitable.*
☒	☐	For Bayesian analysis, information on the choice of priors and Markov chain Monte Carlo settings
☒	☐	For hierarchical and complex designs, identification of the appropriate level for tests and full reporting of outcomes
☒	☐	Estimates of effect sizes (e.g. Cohen's d, Pearson's r), indicating how they were calculated

Our web collection on statistics for biologists contains articles on many of the points above.

Software and code

Policy information about availability of computer code

Data collection	The R package Dada2 (v1.16) is used to process ITS sequence. BLASTn was used to analyze the ITS sequences and FloJo 10.5.3 to analyze the flow cytometer data.
Data analysis	The codes to estimate autotrophic growth rates and to run individual-based simulations are available at Github with DOI (10.5281/zenodo.5944234) (https://github.com/zhenwu0728/Prochlorococcus_Mixotrophy) with detailed instructions under MIT license.

For manuscripts utilizing custom algorithms or software that are central to the research but not yet described in published literature, software must be made available to editors and reviewers. We strongly encourage code deposition in a community repository (e.g. GitHub). See the Nature Portfolio guidelines for submitting code & software for further information.

Data

Policy information about availability of data

All manuscripts must include a data availability statement. This statement should provide the following information, where applicable:

- Accession codes, unique identifiers, or web links for publicly available datasets
- A description of any restrictions on data availability
- For clinical datasets or third party data, please ensure that the statement adheres to our policy

The oceanographic measurements (CTD data, nutrient concentrations and cell counts) have been submitted to the BCO-DMO database under acronym HADFBA (dataset name "EMS photic zone"). Raw ITS sequences are available from GenBank under BioProject PRJNA802335, accessions SAMN25553516- SAMN25553524.

Field-specific reporting

Please select the one below that is the best fit for your research. If you are not sure, read the appropriate sections before making your selection.

☐ Life sciences ☐ Behavioural & social sciences ☒ Ecological, evolutionary & environmental sciences

For a reference copy of the document with all sections, see nature.com/documents/nr-reporting-summary-flat.pdf

Ecological, evolutionary & environmental sciences study design

All studies must disclose on these points even when the disclosure is negative.

Study description	Estimation of the amount of organic carbon taken up and utilized by Prochlorococcus, using a combination of field analyses and mathematical modeling. Field analysis included oceanographic measurements from the Eastern Mediterarnea, including flow cytometry, genetic and nanoSIMS analyses (detailed below) .
Research sample	An oceanographic profile was taken during August 2017 from the Eastern Mediterranean (station N1200, 32.45°N, 34.37°E). Discrete samples from 11 depths were used for cell counts using flow cytometry and for genetic analyses of the picoplankton population structure by amplicon sequening of the ITS. An in-situ bottle incubation was performed using 13C-bicarbonate and 15N-ammonium. Prochlorococcus cells from two of the samples were FACS-sorted and isotopic enrichment was measured using nanoSIMS. The samples for nanoSIMS analysis were chosen based on their depth and (for the 115m sample) the presence of multiple Prochlorococcus populations.
Sampling strategy	Sampling was performed using Niskin bottles. Sample size (number of cells for nanoSIMS analysis) depended on the number of sorted cells available for nanoSIMS analysis (multiple fields, n=45-55 cells/sample). No sample size calculation was performed.
Data collection	CTD data were collected on-board and analyzed post-hoc by DS. During the cruise, a log was maintained by DS and made available as a scanned pdf. Cruise log contains detailed description of time of sample collection for each depth, time to sample fixation, etc. Genetic data were obtained by DA as described in the materials and methods. FACS-sorting was performed by DA, DRR and TLK, nanoSIMS acquisition was performed by AV and cells were analyzed by DA, DRR, TLK with the assistance of AV, FE and MV. Modelling was performed by ZW and MJF, and reanalysis of published time series was performed by DS.
Timing and spatial scale	Sampling was performed on a single cruise on August 7th, 2017. All relevant data are rcorded in the manuscript. Sampling location was 32.45°N, 34.37°E.
Data exclusions	No data were excluded from the analysis
Reproducibility	Multiple depths were analyzed to verify reproducibility of nanoSIMS measurements. Attempts for perform flow-sorting of cells from shallower depths were unsuccessful due to limit of sensitivity of FACS sorter. Results of oceanographic cruises (e.g. flow cytometry, ITS sequencing, nanoSIMS) represent a "snap-shot" of a single timepoint, but were consistent with other studies as described in the text.
Randomization	Not relevant for this study (no distinct study groups).
Blinding	Blinding was not relevant to the study during data acquisition. Analysis of nanoSIMS ROIs was performed blind to the sample.

Did the study involve field work? ☒ Yes ☐ No

Field work, collection and transport

Field conditions	Field work was performed during summer in the oligotrophic Eastern Mediterranean. The water column was stratified and nutriens were depleted. These information are included in the mansucript.
Location	The sampling station was n-1200, 2.45°N, 34.37°E. A full description of the sampling location is found in Roth Rosenberg et al, Environmental Microbiology 2021, doi: 10.1111/1462-2920.15611
Access & import/export	Samples were collected on oceanographic cruise as per common practices. The location sampled is not protected (e.g. not a nature reserve), and no permits were required. Samples for nanoSIMS analysis were sent from Israel to Germany as fixed cells in accordance with all regulations.
Disturbance	The study resulted in no disturbance.

Reporting for specific materials, systems and methods

We require information from authors about some types of materials, experimental systems and methods used in many studies. Here, indicate whether each material, system or method listed is relevant to your study. If you are not sure if a list item applies to your research, read the appropriate section before selecting a response.

Materials & experimental systems

n/a	Involved in the study
[x]	[ ] Antibodies
[x]	[ ] Eukaryotic cell lines
[x]	[ ] Palaeontology and archaeology
[x]	[ ] Animals and other organisms
[x]	[ ] Human research participants
[x]	[ ] Clinical data
[x]	[ ] Dual use research of concern

Methods

n/a	Involved in the study
[x]	[ ] ChIP-seq
[ ]	[x] Flow cytometry
[x]	[ ] MRI-based neuroimaging

Flow Cytometry

Plots

Confirm that:

[x] The axis labels state the marker and fluorochrome used (e.g. CD4-FITC).

[x] The axis scales are clearly visible. Include numbers along axes only for bottom left plot of group (a 'group' is an analysis of identical markers).

[x] All plots are contour plots with outliers or pseudocolor plots.

[x] A numerical value for number of cells or percentage (with statistics) is provided.

Methodology

Sample preparation	Seawater samples were collected and fixed on-board using glutaraldehyde, as described in the text.
Instrument	Flow cytometry was performed using a BD FACSCanto, and FACS using a BD FACSAria.
Software	Data were analyzed using FlowJo
Cell population abundance	Post-sorting cell abundances in test runs to validate sorting purity were 2,120 high vs 240 low fluorescence cells in the high population, and 120 high vs 880 low fluorescence cells in the low fluorescence cells.
Gating strategy	Gating of phytoplankton cells for figure 1 was based on common procedure - chlorophyll autofluorescnece (PerCP-Cy5) vs forward scatter. Pico-eukaryotes were defined based on higher PerCP and FSC, and Synechococcus were defined based on phycoerythrin. The gating for flow-sorting Prochlorococcus cells for nanoSIMS was based on the same criteria. A figure for the gating and sorting strategy is provided in the Supplementary Information.

[x] Tick this box to confirm that a figure exemplifying the gating strategy is provided in the Supplementary Information.
